# The burden of hepatitis B virus (HBV) infection, genotypes and drug resistance mutations in human immunodeficiency virus-positive patients in Northwest Ethiopia

**DOI:** 10.1371/journal.pone.0190149

**Published:** 2017-12-27

**Authors:** Tekalign Deressa, Debasu Damtie, Kevin Fonseca, Shan Gao, Ebba Abate, Shitaye Alemu, Yetemwork Aleka, Mark G. Swain, Guido van Marle, Carla S. Coffin

**Affiliations:** 1 Department of Immunology and Molecular Biology, School of Biomedical and Laboratory Sciences, College of Medicine and Health Sciences, University of Gondar, Gondar, Ethiopia; 2 Provincial Laboratory for Public Health, Calgary, Alberta, Canada; 3 Liver Unit, Division of Gastroenterology and Hepatology, Department of Medicine, University of Calgary, Calgary, Alberta, Canada; 4 Department of Microbiology, Immunology and Infectious Diseases, Faculty of Medicine, University of Calgary, Calgary, Alberta, Canada; 5 Ethiopian Public health institute, Addis Ababa, Ethiopia; 6 School of Medicine, College of Medicine and Health Sciences, University of Gondar, Gondar, Ethiopia; Centre de Recherche en Cancerologie de Lyon, FRANCE

## Abstract

**Background:**

In sub-Saharan Africa, the hepatitis B virus (HBV) and human immunodeficiency virus (HIV) infections are endemic. Although there has been great progress in HIV care, universal HBV vaccination and care is lacking. In this study, we aimed to determine the prevalence of HBV, HBV genotypes, and drug resistance mutations in dual infected cases in a cohort of HIV patients in Northwest Ethiopia.

**Methods:**

A total of 308 HIV-1 positive patients were enrolled into the study and tested for HBsAg in plasma. In HBsAg positive samples, HBV DNA was analyzed for HBV genotype using in-house nested PCR with HBV-specific pre-core / core or surface primers, and for HBV drug resistance mutations (DRMs) in polymerase region. Odds ratio at 95% confidence interval was calculated.

**Results:**

Of the 308 HIV-positive subjects, 62.7% were female, median age 38 years (range 18–68, IQR: 27–49), and the median CD4 count 405 cells/μl (IQR: 75–734). Overall, 94.2% were on antiretroviral therapy (ART) frequently with combinations of Zidovudine (AZT)- Lamivudine (3TC)—Nevirapine (NVP). HBsAg was detected in 5.5% (95%CI 2.95–8.08%) of the study participants, of which the majority were infected with HBV genotype A (7A, 2E, 2D, 1C, 1 G). All HIV/HBV positive cases were on ART with anti-HBV activity (i.e., 3TC) and 3TC associated HBV DRMs (i.e., rtV173L, rtL180M, and rtM204V) were detected in 7/13 (53.8%) subjects.

**Conclusion:**

In this cross-sectional study of HIV-infected individuals, we found 5.5% HBV/HIV co-infected cases. Most were receiving the first generation anti-HBV therapy with a low genetic barrier to resistance, and several carried mutations associated with anti-HBV (3TC) drug resistance. These data underscore the importance of integrating HBV screening to the HIV treatment guidelines for better management and prevention of HBV-related liver disease.

## Introduction

The Hepatitis B virus (HBV) is a major global public health problem. According to the World Health Organization (WHO), more than 240 million people are hepatitis B surface antigen (HBsAg) positive chronic HBV (CHB) carriers and are at risk of developing serious liver diseases such as cirrhosis and hepatocellular carcinoma (HCC) [1, 2]. The prevalence of CHB greatly varies worldwide (0.5%-20%), with the greatest burden of disease in Southeast Asia and Sub-Sahara Africa [2, 3]. Persons living with HIV have a disproportionate high burden of HBV infection because of shared risk factors, transmission modes and endemicity [2–4]. Of 36.7 million HIV-infected people, approximately 10% have CHB. HIV increases mortality from cirrhosis and end-stage liver disease in HBV co-infection [5–7]. The estimated liver-related mortality rate in HBV/HIV co-infected patients was 14.2 per 1000 persons-years, while the rate in HBV or HIV mono-infected ones were only 0.8 and 1.7 per 1000 persons-years respectively [8].

In sub-Sahara Africa, HBV infection status of most HIV patients is unknown. Hence, HBV/HIV co-infected patients are often treated with lamivudine (3TC) as the only anti-HBV active agent. In CHB, treatment with first generation nucleos(t)ide analog (NA) (i.e., 3TC) leads to high rate of drug resistance mutations (DRMs) and virological breakthrough in over 20% of HBV infected patients within the first year of therapy [9]. Furthermore, 3TC resistance can confer cross-resistance to other anti-HBV agents (i.e., emtricitabine, telbivudine and entecavir), which may compromise future anti-HBV and anti-HIV treatment options. Thus, for HBV/HIV co-infection most expert guidelines recommend dual active NA with higher genetic barrier to resistance, (i.e., tenofovir disoproxil fumarate or tenofovir alafenamide with combination Emtricitabine or 3TC) as first line therapy.

In Ethiopia, there is limited information regarding the epidemiology of HBV infection, genotypes, and drug resistance associated mutations in the context of HIV co-infection. Most HBV prevalence estimates were based on studies among pregnant women and blood donors [10–13]. Small cohort studies from the central and northern part of Ethiopia have estimated the prevalence rate of HBV in HIV positive patients at 3.0%- 5.9% [14–16], and suggested that CHB may be a significant health concern.

The World Health Organization recommends screening of all HIV patients for viral hepatitis, vaccination against HBV in non-immune individuals and providing anti-HBV therapy in HBV/HIV co-infected patients. However, universal childhood HBV vaccination was not offered until 2008 in Ethiopia. Although HIV therapy and care is now more widely accessible, it is not standard practice in Ethiopia for HIV-positive patients to undergo routine testing for HBV co-infection. In resource-poor settings like Ethiopia, timely updates of the burden and correlates for HBV among HIV patients can significantly impact prevention and treatment strategies. Therefore, the current study aims to investigate the prevalence, HBV genotypes and HBV drug resistance mutations in a cohort of HIV infected patients in Northwest Ethiopia.

## Materials and methods

### Study setting and population

This cross-sectional study was conducted at an outpatient HIV antiretroviral (ART) clinic at the University of Gondar Hospital. The hospital is a tertiary level teaching hospital that provides in-patient and outpatient medical service to ~5 million people in Northwest Ethiopia. The HIV ART clinic has been available in the hospital since March 2005, and as of 2014, it is estimated that 10,000 HIV+ adults were seen, of which 6000 were on combination nucleoside and non-nucleoside reverse transcriptase inhibitors (NRTI and NNRTI). Testing for HBV and hepatitis C virus (HCV) co-infection is not standard of care. From March -July 2016, a total of 308 subjects, documented HIV antibody positive, were recruited into the study. Subjects were excluded if had end-stage acquired immunodeficiency syndrome (AIDS), multisystem illness, immunosuppression, and severe malnutrition. This study was performed according to the Declaration of Helsinki and received ethics approval from the institutional review board of University of Gondar, and Federal Ministry of Science and Technology of Ethiopia. All subjects provided written informed consent to participate.

### Collection of clinical data and biological samples

A total of 308 laboratory confirmed HIV-infected adults were selected by a systematic random sampling technique in an outpatient setting, and provided the necessary information and samples. The sample size was determined based on a single population formula, considering previously reported 6% prevalence of HBV [16], with 95% confidence interval (CI), 5% margin of error. Variables were chosen for inclusion based on their clinical relevance and included age, sex, marital status, residence, occupation, histories of alcoholism, multiple sexual partner and unsafe injections, and self-reported history of prior HBV and/or hepatitis C (HCV) testing and/or treatment. Available clinical data on date of HIV diagnosis, ART, CD4+ T cell count were extracted through chart review. A total of 8 ml whole blood was drawn for peripheral blood mononuclear cells (PBMC) and plasma isolation using Ficoll-Hypaque gradient centrifugation as described previously [17]. Both plasma and PBMC were shipped to the University of Calgary, Alberta, Canada in dry ice; with appropriate importation and transportation permits from the Public Health Agency of Canada and the University of Calgary occupational health and safety office. Samples were stored at -80°C until analyzed.

### HBsAg testing and HBV sequencing analysis

All plasma samples were tested for HBsAg at the Alberta provincial laboratory for public health using a commercial immunoassay (ARCHITECT; Abbott Diagnostics, Mississauga, Ontario, Canada). In HBsAg positive cases, HBV genomes were detected following total DNA isolation from 500μL plasma and/or 1–2 × 10^6^ PBMC by an in-house nested PCR using HBV surface (S) or core (C) and polymerase (P) gene-specific primers, as previously described [7,18]. All experiments included parallel mock nucleic acid isolations and PCR water negative controls. Recombinant HBV DNA or DNA from serum/PBMCs of known HBsAg positive patients was used as positive controls.

The HBV genotype and drug resistance mutations (DRMs) were determined by direct Sanger sequencing of S, C and P gene fragments. Samples were sequenced in both directions with universal primers at 3730 XL sequencing system (Applied Biosystem, Foster City, CA, USA). The sequences were translated for S, C, and P protein and aligned with sequences of the same genotype to determine putative non-genotypic substitutions. Genbank reference sequences were used for alignment. For all samples, aa 61–250 of the P/RT and aa 100–160 of the S antigenic determinant region were analysed. Genotype was determined with the NCBI genotyping tool (www.ncbi.nlm.nih.gov/projects/genotyping/). Phylogenetic tree was constructed using the Maximum Likelihood method and the Tamura 3-parameter model [19]. The bootstrap values based on a 1000 replicates is shown next to the branches. Neighbor-Join and BioNJ algorithms were used and pairwise distances estimated using the Maximum Composite Likelihood (MCL) approach. All alignments and evolutionary analyses were conducted using MEGA7 [20]. HBV recombinant analysis was performed by jumping profile Hidden Markov Model (JpHMM) [21]. All sequences were submitted to GenBank (accession number pending).

### Data analysis

Data analysis was performed using SPSS (v. 20, SPSS Inc., Chicago, IL). Baseline characteristics of the study participants were reported as percentages or medians with inter-quartile ranges (IQR). Bivariate and multivariable logistic regression analyses were performed to determine the association between variables. Odds ratios and 95% confidence intervals were calculated to determine the strength of association between HBsAg seropositivity and relevant sociodemographic and clinical factors. P-values less than 0.05 were considered statistically significant for all analysis.

## Results

### Summary of clinical and demographic data

Of the 308 HIV positive patients enrolled, 62.7% were female, median age 38 years (range 18–68, IQR: 27–49), and 43% unable to read and write. Most of the participants (94.2%) were on ART, and median CD4^+^T cell count was 405 cells /mm^3^ (IQR: 75–734). The most common ART regimen was combination of Zidovudine (AZT)-lamivudine (3TC)–nevirapine (NVP), followed by TDF-3TC-Efavirenz (EFV). The demographic and baseline characteristics of the study population are summarized in Table 1.

**Table 1:** Sociodemographic and clinical characteristics of study subjects at ART clinic of the University of Gondar Hospital.

Characteristics	Frequency (%), N = 308
**Sex**	
** Male**	115(37.3)
** Female**	193(62.7)
**Age (years)**	
** Median(IQR)**	38(11.0)
** Range (min-max)**	50(18–68)
**Education**	
** Illiterate**	132(42.9)
** Elementary**	64(20.8)
** Secondary and above**	112(36.4)
**On ART**	290(94.2)
**ART compliance**	
** Yes**	272(93.8)
** No**	18(6.2)
**CD4 T Cell count, Median (IQR)**	405 (75–734)
**Platelets Count, Median (IQR)**	269(165–373)
**Rx for Liver disease, n(%)**	21(6.8)
**Family History of liver diseases, n (%)**	7(2.3)

IQR: interquartile range; ART: antiretroviral therapy; Rx: treatment

### Prevalence of HBV/HIV coinfection, HBV genotypes and drug resistance profile

In total, 17 cases (5.5%, 95%CI 2.95–8.08%) tested HBsAg positive (9 male/8 female, median age 39 years (range, 20–60). All 17 cases were on combination ART with anti-HBV activity (12 on 3TC, and 5 on combination of TDF and 3TC) for a median duration of 6 years (range, 2–10). Median CD4^+^ T cell count of the co-infected patients was 493 cell/mm^3^ (range, 83–1065) (Table 2).

**Table 2:** Clinical and virological characteristics of HBV/HIV co-infected patients (n = 17).

ID	Age/sex	HBV genotype	CD4+ T cell^a^	PLT count	Anti-HBV agent	ART duration^b^	DR Mutations	WHO stage
**HP14**	49/M	-	730	531	3TC	7	-	I
**HP22**	38/M	A	224	134	3TC	6	rtV173L, rtL180M,rtM204V	I
**HP32**	60/F	A	583	260	TDF, 3TC	3	rtV173L, rtL180M,rtM204V	I
**HP51**	37/F	A	493	355	3TC	8	rtV173L, rtL180M,rtM204V	I
**HP64**	52/M	A	209	222	TDF, 3TC	4	rtL180M	I
**HP82**	39/M	E	83	234	TDF, 3TC	6	WT	I
**HP95**	40/F	E	640	265	3TC	3	rtV173L, rtL180M,rtM204V	I
**HP106**	39/M	-	712	147	3TC	9	-	I
**HP113**	39/F	C	483	278	3TC	8	rtV173L, rtL180M,rtM204V	I
**HP123**	28/M	A	206	92	3TC	5	rtV173L, rtL180M,rtM204V	I
**HP141**	24/F	A	125	272	TDF, 3TC	2	WT	I
**HP169**	37/F	D	390	377	3TC	9	WT	I
**HP193**	43/M	-	262	184	3TC	10	-	I
**HP248**	30/F	G	619	211	3TC	5	WT	III
**HP258**	35/M	A	666	305	3TC	2	WT	I
**HP259**	35/M	-	1065	234	TDF, 3TC	3	-	I
**HP268**	39/F	D	593	388	3TC	7	WT	I

3TC, Lamivudine; TDF, Tenofovir, PLT, Platelets count (x 10^3^/mL); DR, drug resistance;

^a^CD4+T cell count (Cell/μl);

^b^ ART duration (in year)

We were able to successfully PCR amplify and do sequence analysis of HBV genomes in 13/17 HBsAg positive patients. Phylogenetic analysis of these samples revealed that 53.8% (7/13) of the patients were infected with HBV genotype A, the remaining were genotype D (15.4%), genotype E (15.4%), and others (15.4%) (Table 3). Representative phylogenetic trees of the isolates are indicated in “S1 and S2 Figs”.

**Table 3:** Summary of clinical and demographic characteristics of the 17 HBV/HIV co-infected patients at the University of Gondar Hospital, March-July, 2016.

Variables	N = 17
Sex	
Male	9(52.9)
Female	8(47.1)
Age	
Median, years(Range)	39 (24–60)
CD4 T cell count,	
Median, (Range)	493 (83–1065)
% On ART with anti-HBV, n (%)	
Lamivudine (3TC)	12 (70.6)
Tenofovir (TDF) + 3TC	5 (29.4)
ART duration, year	
Mean (SD)	5.7 (2.6)
Median (Range)	6 (2–10)
Genotype, n (%)	
A	7 (41.2)
D	2 (11.8)
E	2 (11.8)
C	1 (5.9)
G	1(5.9)
Not Determined	4 (23.5)

Analysis of HBV P/reverse transcriptase region sequence displayed multiple variants at residues associated with 3TC drug resistance (i.e. rtV173L, rtL180M, and/or rtM204V) in 7/13 HBV/HIV co-infected patients tested. The most frequent HBV DRM found was rtL180M (53.8%), followed by rtV173L (46.2%), and rtM204V (46.2%). Of note, 5 out of 7 patients with DRMs were receiving 3TC as the only anti-HBV agent (Table 2). However, the classical vaccine escape mutation G145R was not detected.

### Risk factors for HBsAg seropositivity

Binary and multivariable logistic regression analysis was performed to determine sociodemographic and other risk factors for HBV seropositivity (S1 Table). Variables like age, sex, residence, occupation, current CD4+ T lymphocytes count, alcohol abuse, unsafe injection, tattooing, multiple sexual partner, history of sharing sharp objects, WHO stage, HAART status, HAART regimen, and HAART duration were included in the analysis. Patients with a history of sharing sharp objects (razors, and objects for skin piercing) were more likely to be HBsAg positive (AOR 3.4, 95% CI 0.6–19.6), as well as alcohol abuse (AOR 2.3, 95% CI 0.72–7.32), and having multiple sexual partner (95% CI 0.8–8.04, AOR 2.5).

## Discussion

Hepatitis B is common among persons living with HIV owing to their common transmission modes, risk factors, and overlapping endemicity. Co-infections are associated with increased morbidity and mortality than those caused by either HIV or HBV alone [2–6]. Thus, data on the proportion of HBV infection, genotypes and DRMs among HIV patients, especially in HIV high burden settings like Ethiopia, would inform policy makers for prevention, treatment, and control of HBV/HIV co-infection. In this study, we found the prevalence of HBV to be 5.5%, which was very similar with 3.0%-5.9% rates found in previous studies from Ethiopia [14–16]. Yet, it is lower when compared with the 9.7% HBV/HIV co-infection rate in South Africa, 16.7% in Ghana, and 20.4% in Malawi [22–24]. These could be due to differences in the study population and prevailing risk factors. We do not have data to date on HBV core antibody and HBV surface antibody in this cohort, thus it is possible that some of these study subjects could have natural or vaccine induced immunity to the HBV. However, universal vaccination was not introduced in Ethiopia until 2008 and is not yet widely implemented in rural areas.

It is noteworthy that 94% of the study participants were on ART treatment with at least one HBV-active agent for a minimum of six months (median CD4 T cell count was 493 cell/μl). Hence, it appears that with ART restoration of immunity, subjects could have spontaneously cleared HBsAg following recovery from acute HBV infection. In this scenario, these individuals may carry occult HBV infection (i.e., HBsAg negative, anti-HBc and/or anti-HBs positive, with low-level HBV DNA in plasma) [25]. In the current study, we randomly tested 12 HBsAg seronegative samples and observed that 5/12 were positive for anti-HBc (data not shown). This observation suggests that the burden of chronic as well as occult HBV in this cohort could be much higher than the figure in this report, and is an area of further study.

We found a marginally higher prevalence of HBV/HIV co-infection in male patients (i.e., 7.8% vs 4.1% in females). This was in agreement with a number of previous studies [15, 16, 26], although reasons for such gender disparity are unclear. This study also found that subjects with a history of alcohol abuse, sharing objects for skin piercing, and multiple sexual partners had slightly higher likelihood of HBsAg seropositivity [16, 26, 27]. Therefore, it is possible that gender differences in behavioural risk factors could contribute to higher prevalence of HBV co-infection in male patients.

It is recommended that all HBV/HIV co-infected patients receive highly active ART with dual anti-HIV/HBV activity, irrespective of their CD4+ T cell counts. The combination of TDF with 3TC/ FTC is recommended as a highly effective first-line treatment for HBV; but treatment with 3TC as the only anti-HBV agent in ART is not recommended due to the risk of anti-HBV resistance development [28, 29]. In this regard, we found that ~29.4% (5/17) of HBV/HIV co-infected patients in our setting were treated with ART containing 3TC and TDF, and 12/17 was on ART with 3TC as the only HBV-active agent. We found that more than half of these patients, for whom sequence data were available, had 3TC selected DRMs (rtV173L, rtL180M, and rtM204V). This rate is much higher than the 3TC resistance mutations rates (<15%) reported from other sub-Sahara African countries [30, 31]. The discrepancy between our finding and the other African cohorts could be related to differences in time on HAART, the timing of HBV infection in relation to HIV-1 infection and/or to factors related to study design. In light of these, our observation has important implications for current HBV management in HIV patients and underscores the need for screening all HIV patients for HBV prior to initiation of ART.

In this study, we have also determined HBV genotypes circulating among HBV/HIV co-infected subjects to identify the impact of different genotypes on clinical outcomes such as development of 3TC resistance mutations. Currently, about ten HBV genotypes (A-J) have been identified, with genotype A, D, and E circulating in Sub-Sahara Africa [32]. The knowledge of HBV genotypes has gained special attention, because it is essential in predicting HBV infectivity, transmission mode, response to treatment, and disease progression [33–35]. It has been observed that African patients are at risk of aggressive HCC development at a young age, even if non-cirrhotic. This increased HCC risk has been attributed to environmental factors such as the presence of contaminating dietary aflatoxins, and viral factors such as specific HBV genotype (i.e., Genotype A) [36–38]. In the current study, we found that 7/13 of the co-infected patients carry HBV genotype A, but interestingly the others included genotype C, D, E, and G. This finding confirms and expands the previous reports from Ethiopia that reported HBV genotype A to be the predominant genotype circulating in the country [39].

In the current study, all HBV/HIV co-infected patients were on ART with anti-HBV activity and likely had a low level of HBV DNA, hence it was not possible to obtain sequence data in 4 cases found to be HBsAg positive. Additionally, explanatory variables such as family history of liver disease, history of sharing sharp objects, and ART compliance were collected by questionnaire and may introduce the possibility of recall bias. Nevertheless, the gap identified by our study in the current management of HBV in HIV patients may have an implication for the public health policy makers and merits further investigations.

In conclusion, our study found that 5.5% of HIV patients followed in Gondar University Hospital, Ethiopia were HBsAg positive. Most were receiving the first generation anti-HBV therapy with a low genetic barrier to resistance, and several carried mutations associated with anti-HBV (3TC) drug resistance. These data underscore the importance of integrating HBV screening to the HIV treatment guidelines for better management and prevention of HBV-related liver disease.

## Supporting information

S1 Fig Neighbor-joining phylogenetic tree based on P/RT gene from HBV-HIV co-infected Ethiopian patients and reference sequences representing all known HBV subgenotypes available at the GenBank. The bootstrap values based on a 1000 replicates is shown next to the branches. Ethiopian HBV isolates are shown in color.
(TIF)

S2 Fig Neighbor-joining phylogenetic tree based on Pre-CC gene from HBV-HIV co-infected Ethiopian patients and reference sequences representing all known HBV subgenotypes available at the GenBank. The bootstrap values based on a 1000 replicates is shown next to the branches. Ethiopian HBV isolates are denoted as HP followed by numbers.
(TIF)

S1 Table Multivariate analysis of associated factors for HBsAg seropositivity among HIV patients. (DOCX)
