# Supplementary figures and images for: The burden of hepatitis B virus (HBV) infection, genotypes and drug resistance mutations in human immunodeficiency virus-positive patients in Northwest Ethiopia
Source: PLoS One. 2017 Dec 27;12(12):e0190149. doi: 10.1371/journal.pone.0190149 (PMC5744989; doi:10.1371/journal.pone.0190149)

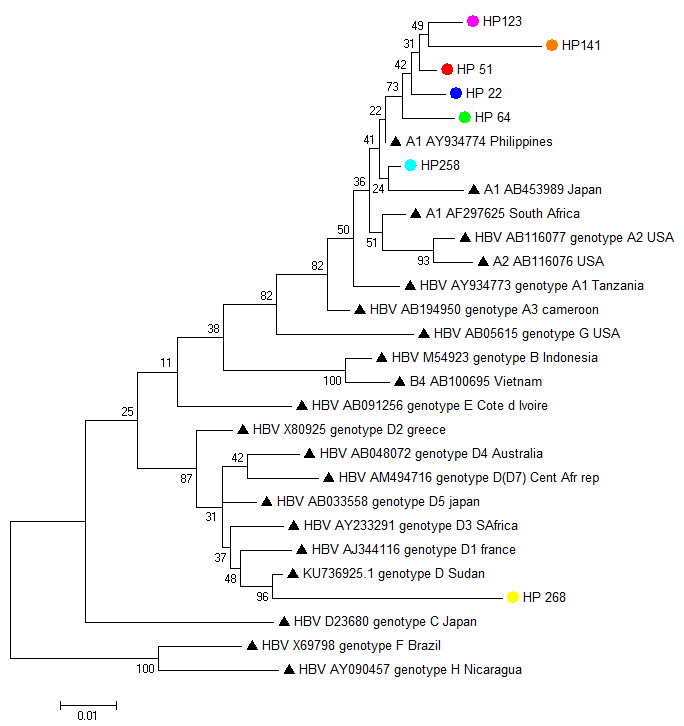

Supplement: S1 Fig — The bootstrap values based on a 1000 replicates is shown next to the branches. Ethiopian HBV isolates are shown in color. (TIF) [file pone.0190149.s001.tif]

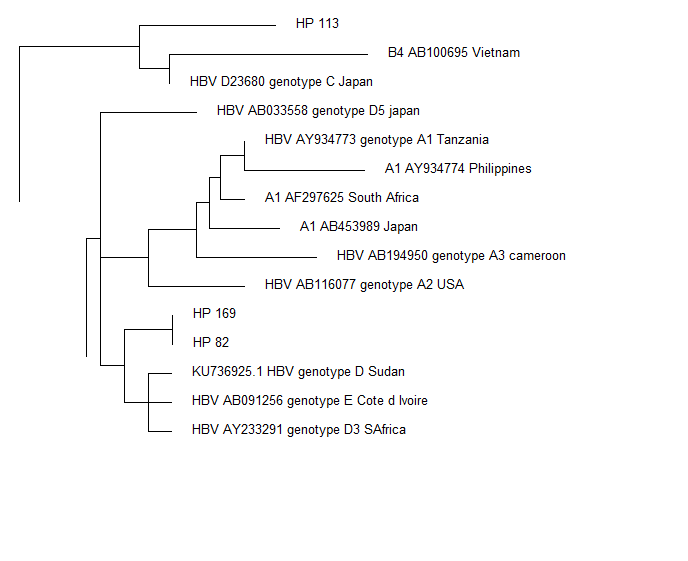

Supplement: S2 Fig — The bootstrap values based on a 1000 replicates is shown next to the branches. Ethiopian HBV isolates are denoted as HP followed by numbers. (TIF) [file pone.0190149.s002.tif]
