# Supplementary material for: The burden of hepatitis B virus (HBV) infection, genotypes and drug resistance mutations in human immunodeficiency virus-positive patients in Northwest Ethiopia
Source: PLoS One. 2017 Dec 27;12(12):e0190149. doi: 10.1371/journal.pone.0190149 (PMC5744989; doi:10.1371/journal.pone.0190149)
Supplement: S1 Table — (DOCX) [file pone.0190149.s003.docx]

**S1 Table. Multivariate analysis of associated factors for HBsAg seropositivity among HIV patients**

| **Variables (Yes vs No)** | **HBsAg Seropositivity** | | **COR(95%CI)** | **AOR (95%CI)** | **P- Value** |
| --- | --- | --- | --- | --- | --- |
|  | Yes | No |  |  |  |
| **Tattooing** | 5(6.5) | 72(93.5) | 1.27(0.43-3.72) | 1.41(0.45-4.36) | 0.56 |
| **Unsafe injection** | 2(5.7) | 33(94.3) | 1.04(0.23-4.76) | 0.52(0.08-3.56) | 0.50 |
| **Sharing sharp objects** | 3(9.4) | 29(90.6) | 1.94(0.25-7.14) | 3.4(0.6-19.6) | 0.17 |
| **Family History of liver disease** | 1(14.3) | 6(85.7) | 2.97(0.94-26.2) | 1.81(0.13-24.75) | 0.66 |
| **Alcohol abuse** | 5(10.6) | 42(89.4) | 2.47(0.83-7.37) | 2.3(0.72-7.32) | 0.16 |
| **Having multiple sexual partner** | 5(11.1) | 40(88.9) | 2.62(0.87-7.82) | 2.5(0.8-8.04) | 0.11 |

CI, Confidence interval; COR, Crude odds ratio; AOR, Adjusted odds ratio
